# The relationship between patient-related factors and medication adherence among Nigerian patients taking highly active anti-retroviral therapy

**DOI:** 10.4314/ahs.v17i3.16

**Published:** 2017-09

**Authors:** Sabina O Nduaguba, Rebecca O Soremekun, Olubusola A Olugbake, Jamie C Barner

**Affiliations:** 1 Health Outcomes and Pharmacy Practice Division, The University of Texas at Austin College of Pharmacy, Austin, Texas, United States; 2 Department of Clinical Pharmacy and Biopharmacy, Faculty of Pharmacy, University of Lagos, Lagos, Nigeria

**Keywords:** Medication adherence, patients, anti-retroviral therapy

## Abstract

**Background:**

Through several initiatives, there are increasingly more people who have access to anti-retroviral therapy. Adherence to therapy is, however, necessary for successful management of disease.

**Objectives:**

The objectives of this study were to describe adherence rates and determine what patient-related factors are related to adherence to anti-retroviral therapy among adult patients in an HIV clinic located in Lagos, Nigeria.

**Methods:**

Adherence was measured using the two-week self-recall method. Barriers, satisfaction with therapy, and socio-demographic and clinical variables served as independent variables. Data were collected via self-administered surveys.

**Results:**

Most of the patients (79.5%) reported 100% adherence. The significant (p<0.05) barriers to adherence were forgetfulness, running out of medication, alcohol use, and medication side effects. For every unit increase in the number of barriers, patients were 60.8% less likely to be 100% adherent (p <0.05, odds ratio, OR = 0.392, 95% CI = 0.295–0.523).

**Conclusion:**

Interventions should target helping patients cope with forgetfulness, specifically employing strategies to overcome busyness in schedules, being away from home, and tiredness.

## Introduction

Adherence, which has been defined by the World Health Organization as the “extent to which a person's behaviour corresponds with agreed recommendations from a health care provider”^1^, is all encompassing ranging from following recommendations on how to use a medication to adjusting one's behaviour and making lifestyle changes. Medication recommendations include the dosing frequency, how long to use the medication, dietary restrictions and other medications to avoid. It is usually expected that if a drug is used according to recommendations, optimal benefits of cure, prevention, or control of disease will occur. Anti-retroviral agents, which are used in the management of Human Immunodeficiency Virus (HIV) infection, do not have the capacity to totally eliminate the virus from an infected individual but will help in achieving virological control if the patient is adherent. Besides achieving virological control, antiretroviral drugs enhance diminished immune function and limit the development of resistant strains of the virus.^2^ Successful medication management of the infection involves taking medication for life with at as high as 95% adherence for virological control.^3^

More than 36 million people are living with HIV globally, with 1.1 million people dying from AIDS-related causes in 2015.^4^ Although the incidence of HIV is declining and more people are gaining access to antiretroviral medication^5^, the benefits of medication cannot be derived unless patients take their medication. Globally, adherence to antiretroviral agents varies between 37% and 83%.^1^

Adherence is a continuous and dynamic process especially in drug therapy of chronic illnesses such as HIV/AIDS and as such patients have to be closely followed.^1^ The consequences of poor adherence to long-term therapies include a reduction in the effectiveness of treatment. For HIV/AIDS, this involves the development of drug resistance, increased risk of transmission of drug resistance to other infected individuals during high risk activity, treatment failure (increased viral load and decreased CD4+ count), disease progression, and death. Because treatment failure is closely associated with non-adherence, factors affecting adherence must be continually assessed and addressed.^6^

The World Health Organization has identified five sets of factors that affect patients' adherence to therapy: socio-economic, health care team and system-related, condition-related, therapy-related, and patient-related factors. These sets of factors including health care team and system-related factors, are mostly dependent on patient characteristics and experiences. For example, a good patient-provider relationship can improve adherence.^1^ In particular, patients' satisfaction with therapy has been linked to adherence.^7^ Since, it encompasses patients' experience with the health-care system, it deserves special attention. Because adherence depends on active participation of the patient, it is necessary that interventions designed for patient populations are mostly relevant for the individual patients.

In Nigeria and other parts of Africa, several studies assessing factors affecting adherence have been conducted.^8^–^17^ Among the studies measuring adherence by self-report, the proportion of ART-adherent patients range from 63% to 88% for optimal adherence and 15% to 73% for full adherence. Charurat and colleagues measured adherence using pharmacy refill rates and found only 26% of the patients in their study to be optimally adherent.^12^ These studies found age, gender, marital status, educational status, personal beliefs about health, traveling distance, duration of time on antiretroviral therapy, CD4 count, forgetfulness, and willingness to disclose status to be associated with adherence. While a lot of attention has been given to patient characteristics and other patient-reported barriers to adherence, little attention has been given to patient satisfaction as a potential factor affecting adherence. The objectives of this study were to describe adherence rates and to determine which patient-related factors (sociodemographic, clinical, adherence-related barriers, and/or satisfaction with medication) are related to adherence to therapy among patients living with HIV (PLWH) receiving ART in a treatment center in Lagos, Nigeria.

## Methods

This study was a cross-sectional survey conducted in the AIDS Prevention Initiative of Nigeria (APIN) clinic of the Lagos University Teaching Hospital, a tertiary center in Lagos, Nigeria. This clinic has been operating since 2007 and is open from 8am to 4pm on weekdays, excluding holidays. It provides Highly Active Antiretroviral Therapy (HAART) free of charge to patients. For acutely ill patients, nurses and peer support patients are available for home visits and medication delivery. As of 2012, the clinic had at least 6,000 patients of varying socioeconomic status who were registered and receiving HAART, typically living between 100m and 1km. Patients receive refills on a monthly basis. Those included in this study were a convenient sample of patients living with HIV/AIDS, 18 years or older, receiving HAART, and had visited the clinic between June and July 2012. Proxies who were picking up medications for a patient were excluded. The study was approved by the Research and Ethics Committee of the College of Medicine at the University of Lagos.

In this study, the main dependent variable was self-reported adherence which was operationalized as the percentage of doses a patient actually took relative to the number of doses required within the past two weeks.^9^ This is mathematically represented as:

$$\frac{\begin{array}{l} \text{Required number of doses per day X 14 - Number of}\\ \text{doses missed X 100\%} \end{array}}{\text{Required number of doses per day X 14}}$$

Using the above formula, patients were categorized as fully adherent (100% adherent) or not fully adherent (< 100% adherent) and as optimally adherent (≥95% adherent) or not optimally adherent (< 95% adherent).

Patients were asked about barriers to adherence using an eight-item (side effects, forgetting, improvement in health, fear of stigma, running out of medication, alcohol, pill burden, and decline in health) scale introduced with the question, ‘Have any of the following prevented you from taking your medication?’ and with ‘yes’ and ‘no’ as possible responses. Six of the items (side-effects, forgetting, fear of stigma, running out of medication, alcohol, and pill burden) were derived from studies conducted in Nigeria that assessed barriers to adherence.^18^ The remaining 2 items were derived from the World Health Organization's document on adherence to long-term therapies.^1^ The number of items that each patient answered ‘yes’ to was summed to create total number of barriers. Satisfaction with therapy was measured with three items on a five-point Likert scale ranging from 1 (very dissatisfied) to 5 (very satisfied). The questions asked were ‘How certain are you that the good things about your medication outweigh the bad things?’, ‘How satisfied or dissatisfied are you with this medication?’ and ‘Taking all things into account, how satisfied are you about being able to cope with side effects?’. Lastly, socio-demographic (age, gender, marital status, educational status, and occupational status) and clinical (length of time since diagnosis and length of time since commencing HAART) data were collected.

Data were collected every work day for a period of one month because the majority of patients returned to the clinic monthly to receive their free HAART therapy. Patients in the clinic were approached by a researcher and invited to voluntarily participate in the study. Those who agreed were given a packet containing a cover letter, a consent form, and the survey. Those who participated in the study returned a signed consent form and completed survey. The survey instrument was self-administered in English and it took approximately 20 minutes to complete. A required sample size of 361 was obtained using Cochran's sample size formula for categorical data.^19^ Thus data were collected until usable responses reached that number.

Descriptive statistics (frequencies and means) were used to characterize the study variables. Pearson's chi-square(dichotomous data) and analysis of variance (continuous data) were used to examine bivariate relationships with adherence and socio-demographic and clinical variables, barriers, and satisfaction. Fisher's exact test (dichotomous data) was employed in place of chi-square analysis where less than 80% of the cells had expected counts more than 5. Logistic regression was used to determine what factors (age, gender, marital status, educational status, occupational status, length of time since diagnosis, length of time since commencing HARRT, number of barriers, and/or patient satisfaction) were related to medication adherence. Missing values were handled using case-wise deletion. The statistical package, SAS 9.4, was used for data analysis. A p-value of < 0.05 was considered statistically significant.

## Results

To achieve the required sample size of 361, more surveys (384) were collected as some of them were unusable as a result of incomplete responses. Table 1 shows the majority of the study sample were between 18 and 40 years (64.9%), female (58.3%), married (64.8%), working (83.8%), and on HAART for at least one year (82.0%) while a plurality of the sample had a college education (46.5%). The vast majority of patients were satisfied (84.8%) with their medication (mean satisfaction score = 4.1±0.8). Additionally, most of the patients (83.3%) had 0 or 1 barrier with mean number of barriers equal to 0.8±1.0. Based on the two-week self-recall, mean adherence was 99.1%±3.1% and ranged between 75% and 100%. The majority (79.5%) were fully (100%) adherent while a larger proportion (92.9%) were optimally (= 95%) adherent.

**Table 1:** Crosstabulation of patient demographic profile, clinical profile, and satisfaction with therapy and 100% adherence^a^

	100% Adherent (N=287)		Not 100% Adherent (N=74)		Total (N=361)		χ^2^
	N	%	N	%	N	%	
**AGE**^b^							
11–30 years	64	22.6	13	17.8	77	21.6	0.543
31–40 years	117	41.3	37	50.7	154	43.3	
41–50 years	74	26.2	17	23.3	91	25.6	
>50 years	28	9.9	6	8.2	34	9.6	
**Total**	**283**	**100.0**	**73**	**100.0**	**356**	**100.0**	
**GENDER^b^**							
Male	122	42.5	28	38.4	150	41.7	.413
Female	165	57.5	45	61.6	210	58.3	
**Total**	**287**	**100.0**	**73**	**100.0**	**360**	**100.0**	
**MARITAL STATUS**							
Not married	106	36.9	21	28.4	127	35.2	1.888
Married	181	63.1	53	71.6	234	64.8	
**Total**	**287**	**100.0**	**74**	**100.0**	**361**	**100.0**	
**EDUCATIONAL STATUS^b^**							
No college education	153	54.1	38	51.4	166	53.5	.173
College education	130	45.9	36	48.6	191	46.5	
**Total**	**283**	**100.0**	**74**	**100.0**	**357**	**100.0**	
**OCCUPATIONAL STATUS^b^**							
Not working	48	16.9	10	13.5	58	16.2	.496
Working	236	83.1	64	86.5	300	83.8	
**Total**	**284**	**100.0**	**74**	**100.0**	**358**	**100.0**	
**LENGTH OF TIME SINCE DIAGNOSIS**							
<1 year	39	13.6	10	13.5	49	13.6	3.082
1–<3 years	86	30.0	19	25.7	105	29.1	
3–<5 years	87	30.3	30	40.5	117	32.4	
≥5 years	75	26.1	15	20.3	90	24.9	
**Total**	**287**	**100.0**	**74**	**100.0**	**361**	**100.0**	
**LENGTH OF TIME SINCE COMMENCING HAART**							
<1 year	54	18.8	11	14.9	65	18.0	4.050
1–<3 years	95	33.1	23	31.1	118	32.7	
3–<5 years	76	26.5	28	37.8	104	28.8	
≥5 years	62	21.6	12	16.2	74	20.5	
**Total**	**287**	**100.0**	**74**	**100.0**	**361**	**100.0**	
**SATISFACTION LEVEL**							
Dissatisfied	11	3.8	8	10.8	19	5.3	5.956
Neither satisfied nor dissatisfied	28	9.8	8	10.8	36	10.0	
Satisfied	248	86.4	58	78.4	306	84.8	
**Total**	**287**	**100.0**	**74**	**100.0**	**361**	**100.1^c^**	

a Adherence was as determined by the 2-week self recall method

b There were missing values for the factor

c Did not sum to 100.0 due to rounding

Table 1 shows cross-tabulations between patient demographic profile, length of time since commencing HAART, and satisfaction profile with absolute adherence. None of the factors (age, gender, marital status, educational status, occupational status, length of time since commencing HAART, and satisfaction with therapy) were associated with 100% adherence.

Table 2 shows the reasons provided by patients for missing doses. Forgetting was the main reason patients missed doses. Among patients who admitted to forgetting to take their medication, 15.3%, 15.3%, and 9.9% respectively reported being busy, being away from home, and being tired as reasons for forgetting to take their medication.

**Table 2:** Cross-tabulation of barriers to adherence and 100% adherence (N=361)^a^

BARRIERS	100% Adherent (N=287)		Not 100% Adherent (N=73)		Total (N=360)		χ^2^
	N	%	N	%	N	%	
**FORGETTING**							
Yes	68	18.9	62	17.2	130	36.1	94.598^b^
No	219	60.8	11	3.1	230	63.9	
**RUNNING OUT OF MEDICATION**							
Yes	22	6.1	15	4.2	37	10.3	10.474^b^
No	265	73.6	58	16.1	323	89.7	
**IMPROVEMENT IN HEALTH**							
Yes	24	6.7	10	2.8	34	9.4	1.938
No	263	73.1	63	17.5	326	90.6	
**ALCOHOL**							
Yes	14	3.9	10	2.8	24	6.7	7.277^b^
No	273	75.8	63	17.5	336	93.3
**SIDE EFFECTS**							
Yes	12	3.3	9	2.5	21	5.8	7.033^b^
No	275	76.4	64	17.8	339	94.2	
**WORSENING HEALTH**							
Yes	8	2.2	3	0.8	11	3.1	8^c^
No	279	77.5	70	19.4	349	96.9	
**PILL BURDEN**							
Yes	7	1.9	3	0.8	10	2.8	7^c^
No	280	77.8	70	19.4	350	97.2	
**FEAR OF STIGMA**							
Yes	5	1.4	4	1.1	9	2.5	5^c^
No	282	78.3	69	19.2	351	97.5	

a Data were available for 360 patients. Adherence was as determined by the 2-week recall method

b Significant at p<0.05

c Fisher's exact test

The overall logistic regression model was significant (Likelihood ratio: 2 = 65.43, p < 0.05) with number of barriers and satisfaction level being significantly associated with 100% adherence (Wald: 2 = 40.22 and 6.82, respectively; p < 0.05). For every unit increase in the number of barriers, patients were 54.8% less likely to be 100% adherent (p < 0.05, odds ratio, OR = 0.452, 95% CI = 0.353–0.578). Patients who were dissatisfied with therapy were 66.9% less likely to be 100% adherent, compared to those who were satisfied (p < 0.05, OR = 0.331, 95% CI = 0.110–0.996). There was no significant difference between patients who were satisfied and those who were neither satisfied nor dissatisfied. Because number of barriers was associated with adherence, a post-hoc analysis (Pearson's chi-square test) was conducted to determine which specific barriers were associated with 100% adherence (Table 2). Forgetting, running out of medication, use of alcohol, and side effects were significantly associated with less than 100% adherence.

## Discussion

This study was conducted to determine HAART medication adherence rate, barriers to adherence, and the relationship between age, gender, marital status, educational status, occupational status, and length of time since commencing HAART, barriers, and satisfaction and adherence. The results indicated that most (92.9%) patients were optimally adherent (i.e., ≥ 95%). A contributing factor to this high rate of adherence may be the setting in which data was collected. Respondents in this study came from a pool of patients who had come to the clinic for prescription refills. This pool are more likely to be more adherent than patients who were missing refill appointments. Such high rates of adherence (88% to 94%) have been observed in other populations within Nigeria and other parts of Africa.^13^,^14^,^17^ However, some studies using patient self-report have recorded optimal adherence rates as low as 63%.^9^ The goal in counselling is usually to encourage the patient to take medication according to directions. Hence, although most patients are optimally adherent (≥ 95% adherence), interventions aimed at bringing other patients to maximally possible adherence levels are still necessary.

Number of barriers and patient satisfaction were found to be significantly associated with medication adherence. Of the barriers investigated, forgetting, running out of medication, alcohol, and side effects were predictors of adherence. As has been found in other studies, forgetting was the major reason for missing doses in this study.^14^,^20^–^22^ This information is important for determining which interventions will work for this population of patients as increasing the effectiveness of adherence interventions may have a far greater impact on the health of the population than improvements in specific medical treatments.^1^ In this study, when patients were asked specifically why they forget to take their medication,fifteen percent reported that it was because of their busy schedule. This might be an indication that some patients see taking their medication as an added burden to their daily activities. This finding is comparable to the study in which some patients regarded the use of anti-retroviral medications as a burden.^23^ Helping patients integrate and incorporate medication taking behaviours in their daily routines may assist them in taking better control of their health and improve medication uptake. Studies show that the use of the pill box and medication reminder systems improve adherence.^15^,^24^ These are relatively in expensive methods for improving patients' organizational skills.

Running out of medication was another reason for missing doses. It may mean that patients are missing refill appointments at the clinic. Medications are free for patients at the clinic. So, investigation is needed to determine whether cost of transportation, clinic hours, or some other reason is responsible. Also, health care counselors need to emphasize the implications of poor adherence, how alcohol use affects adherence, how to manage side effects, as well as stress the lifelong need for adherence. In particular, patients can be encouraged to utilize their social network as high level of social support has been found to be associated with better adherence.^25^,^26^

In our study, satisfaction with therapy was found to be associated with medication adherence. Other studies have found adherence to be associated with satisfaction with different health system components including information received from providers^15^ and services provided.^27^ Based on this, we recommend that periodic feedback be sought from patients and used to tailor care to individual needs and expectations. This may improve patient experience with the health care system and ultimately satisfaction and adherence to recommendations.

Patients were selected using convenience sampling and participation was based on their willingness. As a result, patients who completed the questionnaire may have been systematically different from those who did not, even though data collection was designed to reach all patients over the course of one month. We believe that patient English literacy may have contributed to selection bias because the questionnaire was administered in English and those with little formal education may have been less willing to participate in the study. This may explain why almost half of our respondents had college education. To minimize this limitation, the researcher offered to administer the questionnaire while interviewing those who mentioned that they could not read in English. Adherence using the two-week recall method may have been over-reported due to the need for social desirability by the patients since data was collected at the clinic and researchers were also present during data collection. Patients were, however, reassured that the survey was for research purposes. Also, because adherence was so high, cross-tabulation of adherence and some demographic and clinical variables resulted in small cell sizes, which could make some statistical analyses invalid. To increase the statistical validity of results, some cross-tabulation cells were merged and Fisher's exact test was used in place of chi-square test where less than 80% of the cells had expected cell size less than 5. In addition, while our scale on barriers to adherence was based on a review of the literature, there may have been other barriers that we did not capture. Finally, we had a relatively small sample size for our logistic regression (8 events per covariate). Although some studies indicate that there may be some bias in the regression coefficients, other studies suggest that between 5 and 9 events per covariate, bias is common and where it occurs, it is within acceptable levels.^28^,^29^ However, we still recommend caution in interpreting the results of our study.

In summary, adherence rate among the patients studied at this clinic in Lagos, Nigeria was high. Since having a busy lifestyle may pose a challenge towards patients remembering to take their medication, the use of the pill box, reminder systems, and social support may help improve patients' organizational skills and integrate medication-taking behavior into patients' lifestyle. Tailoring patient care based on patient feedback may improve patient satisfaction with therapy and hence, adherence.
